# How (and why) languages became more complex as we evolved more prosocial: the human self-domestication view

**DOI:** 10.3389/fpsyg.2024.1499994

**Published:** 2025-01-17

**Authors:** Antonio Benítez-Burraco

**Affiliations:** Department of Spanish, Linguistics and Theory of Literature (Linguistics), Faculty of Philology, University of Seville, Seville, Spain

**Keywords:** language evolution, language structural complexity, language uses, prehistory, aggression, human self-domestication

## Abstract

This paper aims to re-examine the problem of the emergence of present-day languages from the specific perspective of the self-domestication account of human evolution. According to this view, our species went through an evolutionary process that parallels the changes experienced by domesticated mammals. Relying on evidence of diverse kind (from paleogenetic to clinical), the paper argues that our self-domestication might have potentiated the cognitive and behavioral features of the human phenotype with an impact on language acquisition and use. Specifically, it might have facilitated the creation of the cultural niche that favors the complexification of languages via a cultural mechanism. The paper further proposes a model of language complexification in the past under the effects of human self-domestication, including the complexification of the structural aspects of language (grammar, prosody, and semantics) and the potentiation of its functional properties (pragmatics). The paper concludes with some suggestions for any future research aimed to test and improve this view.

## 1 Introduction

How language evolved and how present-day languages appeared have been a concern for human beings during millennia. In every human culture, one can find mythological accounts of why humans speak and why they speak the languages they speak. The idea that the emergence of language (and of modern-like languages) represents a true evolutionary leap forward, accounting for the success of the human species, is now widely acknowledged. In their famous paper about the complexification of life on Earth, Szathmáry and Smith (1995) regarded the evolution of language as the two final steps in this process: first, the emergence of a protolanguage without a true syntax, later the emergence of present-days languages, endowed with recursive grammars. Nowadays, language evolution is indeed a favorite topic for many disciplines, not only for linguistics, but also for archeology, paleoanthropology, or genetics, to name just a few. The same can be said of the dynamics followed by languages in our remote past, including the putative type of languages spoken by prehistoric societies, or the patterns of language diversity and change at that time. Explaining language evolution is a formidable task, as language does not fossilize. But it is a doable task now that researchers have massively adopted a truly multidisciplinary approach to this issue. Explaining the dynamics of languages during prehistory is also a great challenge. But it is likewise a doable task now that we have better tools for studying human evolution, and particularly, the changes in the physical environment and in social dynamics in the past. Contributing to this exciting enterprise is the main objective of this paper.

The paper is structured as follows. First, I will provide some background discussion about the relationships between language, languages, and uses of language. This is indeed a long-lasting debate in linguistics, psychology, cognitive science, and allied disciplines, but it is also crucial for understanding language change in the past. The field is progressively moving to more nuanced views of this issue, according to which languages coevolve with human cognition and behavior in response to environmental changes. Against this background, in the second part of the paper, which is the bulk of this contribution, I discuss in detail an original evolutionary model for human language(s) under the view that we evolved increasingly prosocial (aka the human self-domestication hypothesis). The paper finishes with some conclusions and prospects for future research.

## 2 A framework for language evolution studies

Let us begin with some basic clarifications. When we talk about *language evolution* our interest is not put on languages, like Russian, Spanish, or Japanese. It is put instead on our species-specific ability to learn and use these (and many others) languages. More technically, we wish to learn about the evolutionary trajectory of the biological foundations of our species’ ability to spontaneously develop mental rule systems that are put to use in thought and communication. We can call this ability, which essentially equates to Saussure’s *language*, our *faculty of language*. With time, some other denominations have been coined, like *language-readiness* (to stress that this ability mostly depends on our brain), or the one I will use in this paper, namely *human linguisticality*, a recent term proposed by the German linguist Haspelmath (2020). Under this view, our linguisticality is a cognitive ability, so that our attention should be drawn to our biology as our focus of inquiry. By contrast, languages should be understood as the collection of contingent properties of the communication/thought systems that humans eventually acquire as a result of social interactions. If you socialize with Japanese people, you acquire Japanese; if you are born in Spain, you usually acquire Spanish, and the like. Finally, people use their native language (or languages) to fulfil many different functions, like thinking, sharing information with others, socializing, persuading others, playing, and so on. Typically, this entails using your knowledge of the grammar of your language(s) to create utterances that fulfil such functions.

For many years, the mainstream view in the field of (evolutionary) linguistics has been that language can (and should) be construed as a human-specific cognitive faculty which is homogeneous in the species (pathological instances aside) and that resulted from biological changes mostly (e.g., Berwick and Chomsky, 2016, 2017, 2019). Likewise, most linguists agreed that all languages (present-day languages, but also prehistoric languages) are roughly equal in terms of their basic components, fundamental structure, overall complexity, and main functions (e.g., Dixon, 1997: 65–66; Fromkin et al., 2011: 375–374). The reason is that these core features were hypothesized to depend mostly on how our brain is configured and works. So, under this view, once our species emerged, our distinctive brain also emerged, and ultimately, present-day like languages emerged too. Lastly, as noted, people use their native languages to fulfil many functions. But since human behavior and human societies are not so different after all, these functions (at least the basic ones, like socializing, or conveying information) have been assumed to be quite similar worldwide. Accordingly, under this traditional view, causation goes in one direction only: from human linguisticality to languages to uses of language. The approach to language evolution by Noam Chomsky nicely exemplifies this view. According to him (e.g., Bolhuis et al., 2014; Berwick and Chomsky, 2016), language appeared suddenly as a result of one single gene mutation that caused a brain rewiring that brought about recursion, the distinctive feature of all human languages (and of our cognition). Chomsky further argued that this novel brain configuration has not changed since our inception and hence, that it is shared by all human beings (again, pathological instances aside). According to this view, prehistoric languages can be expected to have been quite similar to present-day languages, at least during the last 100.000 years. This refers, of course, to their fundamental properties, since Chomsky acknowledges that all languages do change with time, as when Spanish emerged from Latin. Here, the use of *change* instead of *evolve* stresses that the fundamental properties of languages are expected not to change historically, and accordingly, that the historical change of languages has no impact on the evolution of our linguisticality (see Mendívil-Giró, 2019 for discussion).

Over the years, however, this view has attracted increasing criticism. To begin with, language features can impact on our cognition. For instance, people speaking languages with an object-verb word order, like Japanese, are better at recalling initial items from a list, whereas speakers of final-object languages, like English, are better at recalling the last items from a list (Amici et al., 2019). A reason is that objects are usually a focus of attention and typically, the most informative part of a sentence. Hence, the language we speak conditions, even if subtly, the way in which we perceive the world and process and storage information about the world. More generally, planning to talk also biases our perception and the way in which we process data, because we need to accommodate the structural features of the language we are using. Still, this effect can be more profound, if the habitual encoding and use of such specific language features results in non-linguistic representational and even behavioral effects. Ultimately, aspects of languages that are more costly to process and learn might favor the creation of “cognitive gadgets” through permanent modifications in learning and data-acquisition mechanisms (Heyes, 2018). For example, one could argue that the cognitive device we use for reading is one of such gadgets. Contrary to language, we did not evolve for reading, but “parasite” instead several neuronal devices fulfilling other functions, most notably, the visual word form area, which recognizes visual patterns, as well as the phonological loop, involved in using sounds for conveying meanings (Dehaene and Cohen, 2007; Wandell and Le, 2017). Potentially, these cognitive gadgets could be “fixed” through, e.g., epigenetic inheritance, but this process takes time. Since it also takes time for such cultural innovations to spread and consolidate, these feedback effects posit a challenge to hypotheses arguing that language evolved abruptly.

Likewise, ample research suggests that languages are sensitive to the environment in which they are spoken. Quantitative approaches to phonological diversity have found, for instance, significant correlations between the degree of vocalism and tree coverage (Maddieson and Coupé, 2015). Accordingly, languages spoken in areas that are rich in forests exhibit a higher proportion of vowels, whereas languages spoken in open areas have more consonants. This is seemingly because sound propagates differently in different physical environments: also animals adapt their calls to the medium in which they live (Ey and Fischer, 2009). Interestingly too, tonal languages like Chinese or Thai are usually found in tropical and subtropical regions. This uneven distribution is seemingly explained by the perturbations of phonation caused by desiccated ambient air, as typically experienced in drier and colder regions, which make tonality less efficient for conveying linguistic information (Everett et al., 2015; Roberts, 2018). To offer a last example, recent research has also found a positive correlation between sonority and local temperature, so that languages spoken in cold regions have on average more plosive and fricative sounds, whereas languages spoken in warm areas show more sounds with high sonority, like trills or nasals (Wang et al., 2023).

Not surprisingly, the effects of the social environment on language features are stronger. We are familiar with the impact of different social factors on linguistic diversity within a language. Hence, structural and functional differences can be found between the varieties of a language spoken by children vs. adults, by men vs. women, and the like. It is also widely acknowledged that the context of a conversation (who is speaking, what they speak about, what they speak for, and the like) also affects the structure and the pattern of usage of a language. Nonetheless, the effect of these factors on language features of interest from a typological perspective is more controversial. Most linguists would agree that the vocabularies of the world languages differ because vocabularies store relevant cultural features, which diverge from one society to another (Evans, 2003; Sharifian, 2014; Majid, 2015). Also, languages tend to grammaticalize, in different ways and to different degrees, aspects of the environment in which they are spoken. Quantitative approaches to this issue suggest that many grammatical features can be significantly affected by social factors. For example, the index of agglutination (that is, how complex a word is) negatively correlates with population size (Lupyan and Dale, 2010). Over time, diverse sociological, political, and cultural factors have been suggested to impact on the structure of languages, including the number of speakers, the degree of bilingualism, the tightness or the looseness of the social networks, the sociopolitical organization, or the number of adult learners of a language (Wray and Grace, 2007; McWhorter, 2011; Trudgill, 2011; Nettle, 2012; Sampson et al., 2009). A recent study using nearly 100 morphological and syntactic parameters from the World Atlas of Language Structure (WALS), as well as a dozen of cultural and sociopolitical features of human societies retrieved from D-Place, Ethnologue and Glottolog, has found evidence of an inverse correlation between morphological complexity and sociopolitical complexity, as well as a direct correlation between syntactic complexity and sociopolitical complexity (Chen et al., 2024), in line with the view that languages adapt to their social environment (Lupyan and Dale, 2016).

When one considers all the social factors with an impact on language structure together with the language features subject to variation, an interesting pattern emerges. On the one side, the languages spoken by isolated human groups living in small, close-knit communities with high proportions of native speakers usually exhibit larger sound inventories and complex phonotactics, opaque morphologies (with more irregularities and morpho-phonological constraints), limited semantic transparency (with abundance of idioms and idiosyncratic speech), reduced compositional structure, and less sophisticated syntactic devices. On the contrary, large and complex social networks, involving greater rates of inter-group contacts and cultural exchanges, seemingly favor languages with expanded vocabularies and increased syntactic complexity (including greater reliance on recursion). These languages also exhibit greater compositionality and enhanced semantic transparency, as well as simpler sound combinations and more regular morphologies. Overall, the difference between these two types of languages seemingly results from their differential context-dependency. In fact, the same pattern can be expected for different varieties of the same language, as the standard vs. the vernacular. And of course, this difference can be safely expected to be a matter of degree. In Chen et al.’s paper, (2024) the poles of this continuum are characterized, respectively, as esoteric (or S) languages and exoteric (or X) languages, but as noted, one can expect that esoteric and exoteric varieties of the same language, or more generally, esoteric and exoteric types of communication do exist. Linguistic esotericity is thus related to people sharing considerable amounts of knowledge, whereas linguistic exotericity involves using language in decontextualized settings.

Research on other domains of linguistics beyond language typology and sociolinguistics have converged onto this idea that language structure is sensitive to the environment (and particularly, to the social environment), to the extent that even core language features (that is, features thought to be universal and imposed by our cognitive hardware) can result from language learning and use. This is a robust conclusion, for instance, of studies using artificial grammars and involving iterated learning. In these experiments, core properties of language, like morphology, arise from a trade-off between pressures for compressibility and expressivity (Kirby et al., 2015). Compressibility is the tendency to capture systematic regularities in the form of abstract rules. For example, using the same prefix for all the words with a negative meaning, as in *impossible, improbable*, and the like. Expressivity is the capacity of providing a unique and unambiguous signal for every meaning, as with a list of proper nouns. Less compressible languages (like dictionaries) are more expressive, but more costly to learn. Conversely, compressible languages (like languages with a grammar) are easier to learn, but can incur ambiguity. Accordingly, speakers prefer compressible languages, whereas receivers favor expressive languages. As the relative strength of these two pressures typically changes from one social context to another, different social contexts can be expected to result in different language types endowed with different design properties.

Likewise, studies dealing with recently emerged sign languages reinforce the view that language structure, and even key design features of human language, can be sensitive to the social environment and ultimately, result from cultural evolution. In a language like Al-Sayyid Bedouin Sign Language (ABSL), core features like phonology, word order, or even recursion, develop with time in response to environmental triggers, like the kind and amount of input, the size of the community, or the degree of interaction among speakers (Sandler et al., 2005). Interestingly, this parallels what has been found in some oral languages. The celebrated Pirahã language could be a good example. According to the description of the language by Everett, Pirahã lacks recursion in the domain of complex sentence (Everett, 2005), with this reduced grammar complexity resulting from cultural constraints. These findings open the door to using our current knowledge of the social dynamics in the past for inferring basic aspects of the grammars of the languages spoken during prehistory, that are far beyond the limits of linguistic reconstructions as achieved by traditional methods in historical linguistics.

To complete the picture, one could also expect that social dynamics impact on human cognition (and ultimately on language) either directly or indirectly, through their impact on language structure and use, as sketched above. As for the direct effect, Dunbar (1998, 2009) has argued that the human brain increased in size as we evolved more prosocial and human groups grew larger. Dunbar has further claimed (in, e.g., several of the chapters he authors in Dunbar et al., 2014) that a more sophisticated language capacity (and particularly, advanced storytelling abilities) might have favored the creation of these larger and more complex human groups, since narratives help to reduce social stress. Accordingly, while primates rely on grooming for managing social conflicts, humans have circumvented the limitations of grooming, which is more time-consuming (particularly, in the case of the big social groups we form), and use instead language to resolve conflicts and reinforce bonding. Like grooming, storytelling (but also other activities governed by language, such as feasting or religion) triggers the endorphin system and increases affiliative behaviors. To offer another example, changes resulting in increased joint attention or increased cooperation as human groups evolved larger and more complex can be expected to have improved our pragmatic and conversational abilities, in turn making grammar more sophisticated (see Ferretti, 2022 for details). As for the indirect effects of social dynamics on cognition through their impact on language structure and use, one could hypothesize, for instance, that people speaking S-languages exhibit a potentiated declarative memory compared to their procedural memory abilities. The reason is that the former is typically implicated in vocabulary learning and irregular phenomena across language domains, and it is thus most associated with memorized, opaque, formulaic chunks of language (for instance, idioms and proverbs), which are all more abundant features in this type of languages. By contrast, procedural memory is typically (although not exclusively) implicated in compositional, automated, rule-governed dimensions of language, which are all aspects found potentiated in X-languages (see Benítez-Burraco et al., 2022, and Chen et al., 2023 for more details). Nonetheless, other cognitive differences between the speakers of S and X-languages could be hypothesized to exist too, including differences in working memory, executive function, episodic memory, perception, emotion, or sensorimotor aspects of language.

To finish, the effect on cognition (via its impact on language structure) of the social aspects we are considering here (like network complexity, contacts with other groups, or ways of life) could also result from different social environment potentiating different uses or functions of language. For example, marking identity through language or using language for socializing (as in greetings) can be more important for close-knit groups, whereas sharing de-contextualized information and know-hows with strangers can be more familiar for speakers of X-languages. The structural features of the type of language used to fulfil these functions can be remarkably different. Commands and greetings typically involve short utterances, if not single words, whereas explanations about how the Solar System evolved, as found in a book of astronomy, typically demand long sentences with embedding, passives, and the like. Ultimately, favoring some language functions over others because of sociopolitical reasons can result not just in favoring some structural features of language over others, but also in cognitive differences, since different language functions impose different cognitive demands and entail different patterns of language processing by the brain, also because of the structural differences mentioned above. Typically, referential uses of compositionally-complex language recruit brain areas around the classical language network, which is mostly left-lateralized (Friederici et al., 2017), whereas less compositional linguistic items, like idioms, which are more frequent in informal, emotionally-charged uses of language, involve bilateral activation patterns of the language areas, with the recruitment of right areas (Hertrich et al., 2020). This is also true of figurative language, implicit meanings, background knowledge, discourse contexts, and pragmatic interpretations (Ferstl et al., 2008).

**Figure 1:**
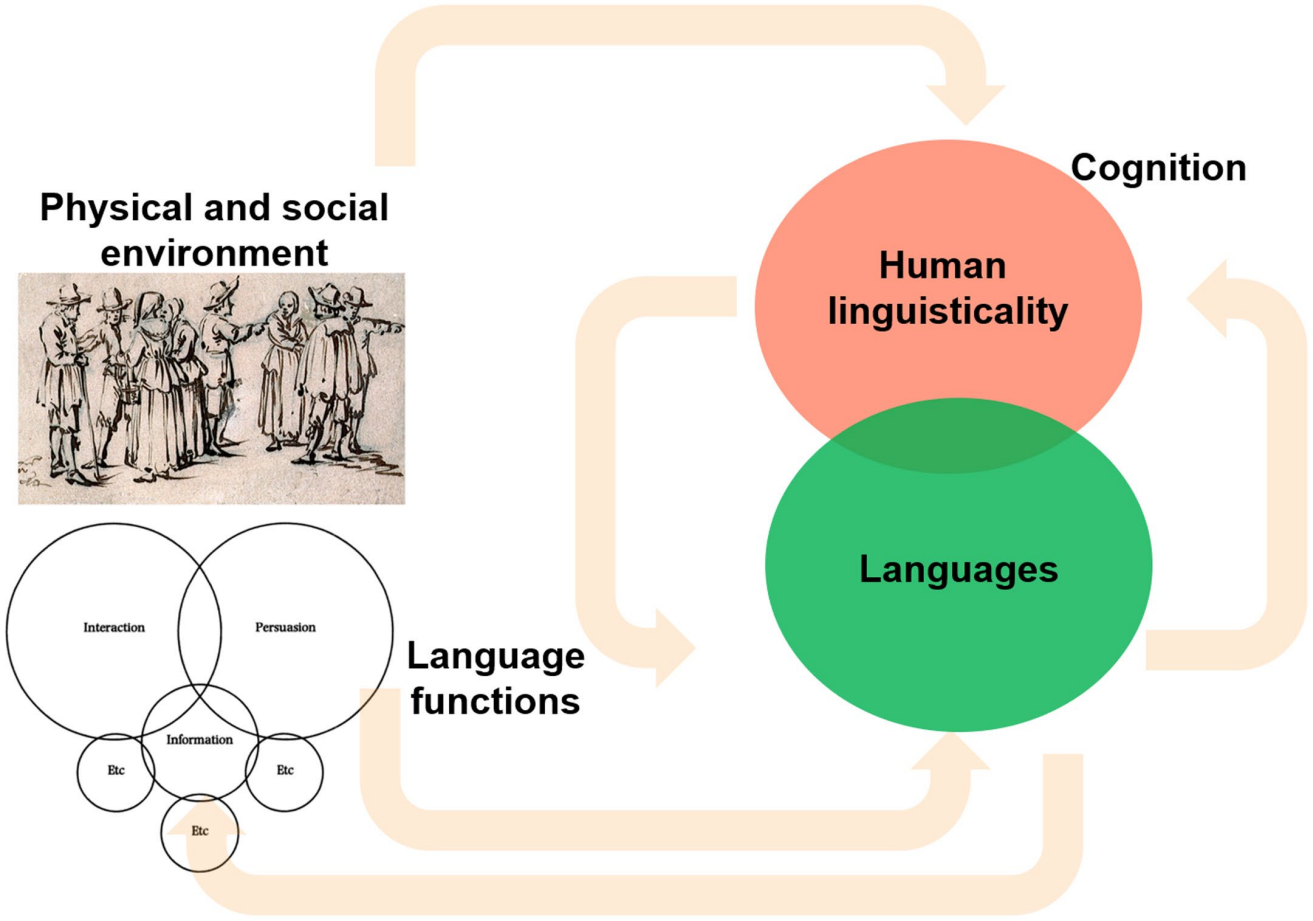
The expected links between human behavior and cognition (including our linguisticality), the languages we speak and the uses we give to them, and the physical and the social environments in which we live (own elaboration).

In summary, in sharp contrast with previous views of the emergence of modern languages as a direct outcome of brain changes, the complex links and feedback effects reviewed in this section between our cognition, our linguisticality, our behavior, the languages we speak, the uses we give to our languages, and the physical and the social environments in which we live (Figure 1), are suggestive of a different evolutionary scenario for present-day languages. Accordingly, one can expect that selected changes in our cognitive architecture and our behavior certainly improved the structural and functional properties of the languages spoken by archaic humans. But at the same time, our languages were certainly shaped by changes in our environment broadly construed, this including our physical environment, but particularly, the type of societies in which we lived, and the cultural niches we created. And to some important extent, these changes also impacted on our cognition and behavior, in turn affecting language structure and use.

The latter is a gradualist scenario for the emergence of modern languages. Compared to saltationist views, it is more in line with the gradual evolution of the human body and human behavior, as attested by paleoanthropological and archaeological research. For instance, there is evidence of a progressive globularization of the human skull/brain, with first archaic humans exhibiting anatomies (and presumably, functionalities) similar to those of late Neanderthals (Neubauer et al., 2018; Gunz et al., 2019). Since a globular brain has been related to our distinctive linguisticality (Boeckx and Benítez-Burraco, 2014), these progressive changes can be expected to have impacted on our language faculty, and ultimately, on the languages we spoke in our remote past. Likewise, evidence of modern behavior (like the use of pigments or ornaments, complex hunting strategies, advanced lithic technologies, or intentional burials) have appeared quite recently only (e.g., McBrearty and Brooks, 2000), with these behavioral innovations being suggestive of some cognitive changes too (Langley and Suddendorf, 2022), and with both types of modifications, cognitive and behavioral, impacting on our language abilities, as previously discussed. It is thus not speculative to hypothesize that more complex languages might have emerged in late prehistory in response to the biological and particularly, the cultural changes experienced by our species. The evolutionary framework of human self-domestication (HSD), which will be examined in detail in the next section, can successfully accommodate this progressive evolution of the human body, behavior, and culture, and emerges as a promising framework for the evolution of language as well.

## 3 Evolving more complex languages as we evolved more prosocial: the self-domestication view

In brief, HSD refers to a recent hypothesis about how our species emerged. It claims that the human distinctiveness is, to a large extent, the outcome of an evolutionary process similar to animal domestication (see Hare, 2017, or Hare and Woods, 2020, for an overview). In animals, domestication is usually triggered by selection for tameness, and in most cases results in a constellation of distinctive traits that are physical, cognitive, and behavioral by nature: the so-called domestication syndrome. It has been suggested that this is because tameness reduces the input to the neural crest, an embryonic structure that gives rise to many different body parts (Wilkins et al., 2014). Certainly, this view of animal domestication is not uncontroversial, mostly because not all domesticates show the whole suite of features purportedly encompassing the syndrome (see Sánchez-Villagra and van Schaik, 2019 or Lord et al., 2020 for critical views), but also because not all experts on animal domestication would agree that these traits result from the hypofunction of the neural crest (see Sánchez-Villagra et al., 2016 and Lord et al., 2020 for some criticism). That said, the hypothesis of HSD builds on the finding in humans of many of the traits commonly observed in domesticated varieties of mammals, including reduced skulls/brains, childish facial features, less hair, prolonged childhood, more time devoted to play, and particularly, a less aggressive behavior (Shea, 1989; Leach, 2003; Somel et al., 2009; Zollikofer and Ponce de León, 2010; Plavcan, 2012; Fukase et al., 2015; Stringer, 2016). Different factors might have triggered HSD: the rise of community living, the advent of co-parenting, changes in our foraging ecology, climate deterioration, and/or the colonization of new territories (see Pisor and Surbeck, 2019; Brooks and Yamamoto, 2021; Spikins et al., 2021; Raviv et al., 2023 for recent discussions). These factors would have promoted a selection toward less emotionally reactive partners and toward tolerance for extra-group individuals, resulting in increased cooperative behaviors. In turn, the hypothesis follows, the behavioral, cognitive, and even physical changes brought about by HSD would have promoted the emergence of many human distinctive features, including our enhanced social cognition, increased cooperation and extended social networks, and ultimately, our advanced technology and sophisticated culture.

Nonetheless, it was the finding that in some birds, domestication results in more complex communicative signals (e.g., Takahasi and Okanoya, 2010; Okanoya, 2017), that paved the way toward claims that HSD could be valuable in capturing key aspects of the evolution of language, specifically, those resulting from cultural evolution. If one recalls the complex interactions discussed in Section 2 between human cognition, human linguisticality, the languages we speak, and our physical and social environment, one could argue that HSD might have brought about both some of the physical, cognitive and behavioral changes with an impact on language structure and use, and the richer interactional niche favoring the complexification of language via a cultural mechanism. Regarding the physical changes, some of the modifications occurred in the human skull/brain and face during the last 100.000 years (as described by, e.g., Cieri et al., 2014), all with a potential impact on language, do resemble the changes in brain size or the snouts of domesticated animals, which typically show smaller brains and less prominent jaws. Likewise, the pigmentation changes usually associated with domestication could account for our distinctive white sclerae, which favors joint attention and face-to-face interactions (Wacewicz et al., 2022). The cognitive and behavioral changes are far more important for the complexification of language, as we discuss in depth in the subsections below. There, we sketch an evolutionary model for language under the forces of HSD. In truth, we expect this model to apprehend the effects on language structure and use of our contrasted trend toward a more prosocial behavior, even if the HSD hypothesis eventually turns to be incorrect as such. The main reason is that in most scenarios, increased contacts between individuals can be safely expected to trigger the sort of behavioral and cognitive changes discussed below.

### 3.1 HSD and grammar

**Figure 2:**
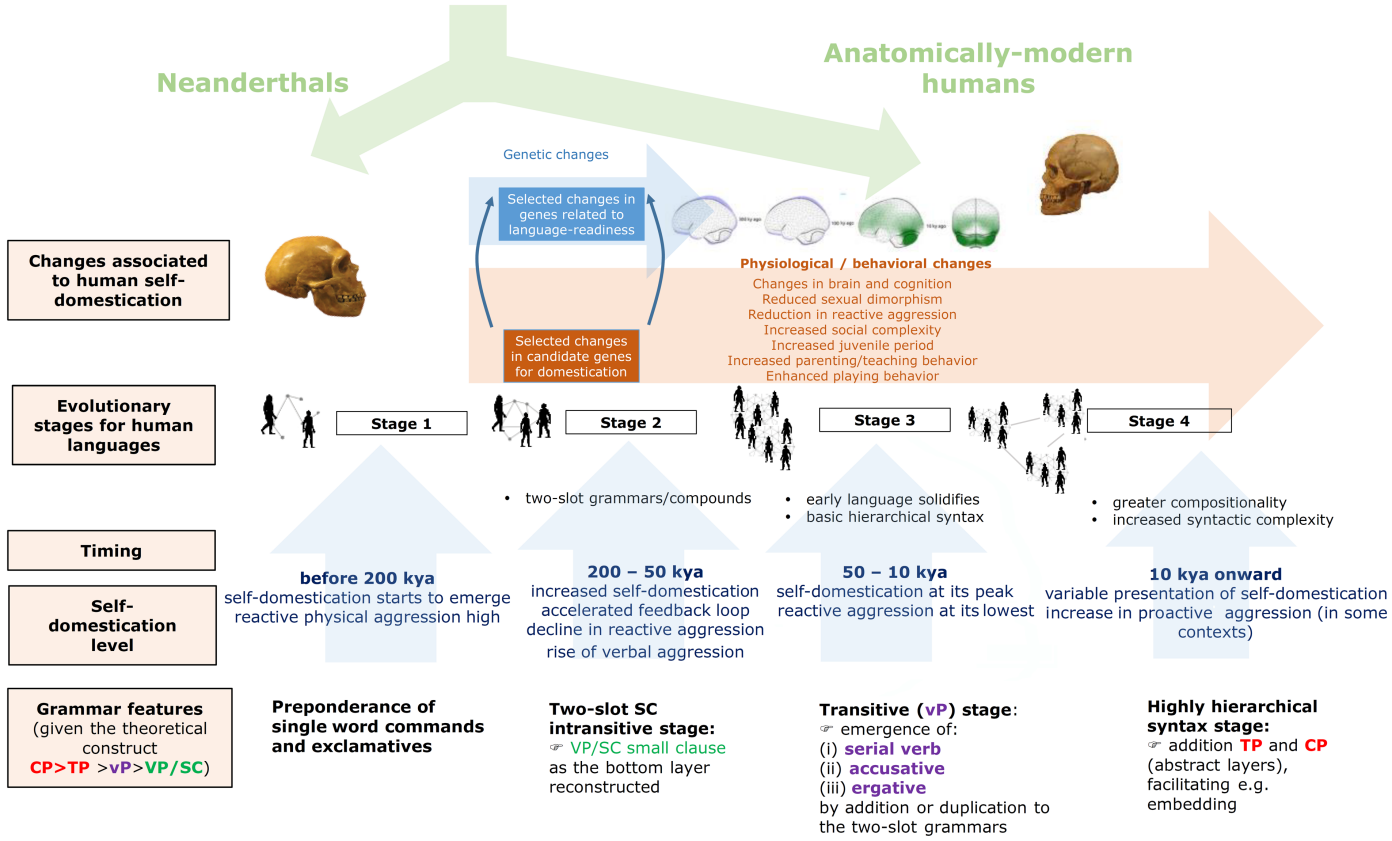
An outline of the evolution of grammar under the effect of HSD (reproduced from Benítez-Burraco and Progovac, 2020, Figure 1).

Figure 2 shows an outline of a model of grammar evolution under the effects of HSD (described in detail in Benítez-Burraco and Kempe, 2018; Progovac and Benítez-Burraco, 2019; Benítez-Burraco, 2020; Langley et al., 2020; and particularly, Benítez-Burraco and Progovac, 2020). As noted, the process encompasses four stages.

Stage 1 corresponds to the beginnings of HSD, roughly 300 kya (thousand years ago). Because reactive aggression was still high, communication through language could not have involved patient and cooperative turn-taking, using long utterances, as with modern languages, but just single-word commands, threats, and exclamations, mostly aimed to convey emotions. Notice that this is a characterization of the human languages purportedly spoken during that period, not a depiction of human linguisticality at that time. Even today, people can rely on simpler linguistic structures/systems in some circumstances, as observed in pidgins or restricted languages, but particularly, when they are angry and involved in verbal disputes.

As features of HSD increased, the cultural process that makes languages more complex seemingly increased too. Hence, reduced reactive aggression would have facilitated the establishment of stronger in-group networks, involving more diverse, frequent, and prolonged contacts between members. All these are factors that make language structure and language use more sophisticated. However, the potentiation of our language abilities, and particularly, the complexification of grammar, might have resulted not only from a cultural process like this, but also from selected brain changes brought about by HSD, as advanced above. One important reason is that the brain regions involved in the control of aggression are functionally connected to, or are in some cases partially overlapping with, the areas that are involved in language processing (see Miller et al., 2008 for discussion). Evidence of this is that the abnormal processing of language cues can result in misperceptions of emotional contents that trigger reactive aggression responses (Miller et al., 2008); and conversely, the activation of aggression responses can inactivate selected language areas, giving rise to abnormal language production and comprehension (Barratt et al., 1997). More specifically, there is evidence supporting the view that this increased control on aggressive responses would have been achieved, specifically, by enhancing the connectivity of the subcortical components of the circuit of aggression to selected cortical areas (see Benítez-Burraco and Progovac, 2021 for details). As discussed by, e.g., Lischinsky and Lin (2020), controlled aggression responses (as in learned aggressive actions) result from an increased control of the hypothalamus (part of the ‘core aggression circuit’) and the striatum (part of the ‘learned aggression circuit’) by the prefrontal cortex. However, the striatum is also a core component of the procedural memory system, and more generally, of the cortico-subcortical networks involved in grammar processing (Murphy et al., 2022). Therefore, it can be hypothesized that HSD enhanced this sort of functional connections, and even partial overlaps. In brief, the more cortical control of aggressive responses as HSD increased, the more potentiated language processing abilities and the more sophisticated grammar… but also the richer cross-modal thought, as cross-modality also demands more connectivity between distant cortical and subcortical areas. As highlighted by many cognitive scientists, most notably Spelke (2003), human cognition excels at this ability of unifying and combining conceptual units belonging to different core knowledge systems. One intriguing possibility to be explored in the future is that both grammar and cross-modality (underlying, e.g., figurative uses of language, as in metaphors) depend on the same combinatorial ability and that this ability can be equated to the basic combinatorial operation in natural languages, which is called Merge by Chomskyan minimalism (Chomsky, 1995).

Returning to the model, this sort of cognitive and behavioral changes might have favored the transition to Stage 2, which might have spread between 200 kya and 100 kya, when the Last Glaciation began. For this stage, single word utterances might have started to be combined in a pair-wise fashion, leading to rudimentary two-slot grammars that would have employed nouns and verbs to express predications. An important use of these early grammars might have been the creation of derogatory compound expressions, which allowed to replace physical (reactive) aggression by verbal aggression (see Progovac and Benítez-Burraco, 2019 for details). In turn, this might have contributed to accelerate HSD, because of the common neurobiological mechanism supporting these three core dimensions of language: aggression, language processing, and cross-modality, as depicted above.

Around 100 kya, climate deteriorated notably and HSD exacerbated, reaching its peak around 50 kya as evidenced by the paleoanthropological record (Cieri et al., 2014). Increased cooperation would have enabled humans to survive during the Glacial Ages. This is Stage 3 in the model. The extremely low levels of reactive aggression during this period likely facilitated more frequent and more diverse contacts between children and adults, resulting in enhanced opportunities for teaching and learning. Increased HSD would have potentiated as well neotenic features in our species, this resulting in prolonged learning periods and increased play behavior. These are all factors that make language more complex, as noted enough. As a result, more sophisticated forms of grammar might have generalized, specifically, the first hierarchical grammars expressing transitivity. The most confident proxy of the languages spoken during Stage 3 are the languages of present-day hunter-gatherer human groups, and more generally, the Type S-languages discussed in Section 2.

As population size increased in response to cultural innovations and climatic changes, inter-group contacts generalized and extensive social networks emerged, relevant for trading and mating. Consequently, the necessity of exchanging information and know-hows with strangers also increased. This probably favored the advent of the second type of complex languages discussed in Section 2, namely, Type X-languages. This is the Stage 4 in the model, whose starting point it is tentatively situated 10 kya, during the transition period from the Paleolithic to the Neolithic. The advance of X-languages can be linked as well to the emergence of new forms of aggression, specifically, proactive (that is, premeditated) aggression, that became generalized during this period (see Wrangham, 2018, or Sarkar and Wrangham, 2023 for discussion). Since X-languages seem quite fit for conscious planning, they could have contributed to large-scale hostilities and escalated battles, and ultimately, to the emergence of cultural institutions around war and peace in complex societies (see Kissel and Kim, 2019 for a general discussion; see Meijer, 2024 for a HSD view). Neurobiologically, being a form of conscious aggression, proactive aggression demands even more control of the circuits of aggression by the cortex (Zhu et al., 2019, 2022), similarly to sophisticated syntax, a hallmark of Type-X languages. Another factor that might have contributed to the emergence of this type of languages was the increased number of people learning them as a second language. Whereas morphological complexity and morphological irregularities seem to be easy for children (allegedly, because they entail a lot of redundancy), they posit a learning problem for adults, who tend to simplify language morphology (and to compensate the less redundancy with their better pragmatic abilities and their more extensive general knowledge) and to potentiate the syntactic aspects of language (since they have more working memory resources) (see Dahl, 2004; Gil, 2009; Lupyan and Dale, 2010, Atkinson et al., 2018 among many others for further discussion).

In summary, this model of grammar change under the effects of HSD ties the different stages in the evolution of morphology and syntax with changes in the management of aggression, either reactive or proactive, and ultimately with the behavioral and cognitive changes brought about by HSD, with both aspects, namely, language features and HSD, being engaged in a mutually reinforcing feedback loop, since both aspects depend on, and impact on a common neurobiological substrate.

### 3.2 HSD and phonology

In a recent paper (Benítez-Burraco and Elvira-García, 2023), we have reasoned that this HSD framework could account as well for some other expected changes in the structure of human languages, particularly, in the domain of phonology. Phonology is essentially tied to speech; hence its evolution has been usually explained in terms of changes in the anatomy of speech organs (e.g., Barney et al., 2012; Conde-Valverde et al., 2021) and their control by the brain (e.g., Fitch et al., 2016; Brown et al., 2021). However, phonology is not totally detached from grammar. For instance, languages typically exhibit diverse morphophonological constraints. Also, phonological processes like ablaut or reduplication are commonly used for expressing grammatical features, like plurality or aspect. Our main hypothesis is that prosody, specifically, might have become complexified in parallel (and possibly, in a positive feedback loop) with the complexification of grammar in response to our increased HSD.

Diverse evidence supports this view:

- Although, as noted enough, HSD is more about changes in human interaction patterns than about major modifications in our bodies, HSD can be expected to have affected, even if subtly, the biological infrastructure of speech, with some potential impact on human prosody, since a main target of domestication is the craniofacial area (Geiger et al., 2022).
- As also noted at the beginning of this section, domestication is known to promote the complexification of sound signals in many species, particularly, birds.
- In all cultures, prosody is used for conveying emotional contents and for coordinating with others; as also noted enough, HSD is expected to have impacted both the control of emotions (via its effects on aggression management) and the way in which we socialize with others (via its effects on interactional patterns between people).
- A neurobiological link between (the control of) prosody and (the control of) aggression can be safely expected. Hence, whereas prosodic anomalies resulting in misperceptions of emotional contents can produce impulsive outbursts, it also happens that a reduced control on aggressive responses impacts negatively on the processing of prosodic cues (see Miller et al., 2008 for details). In truth, the brain areas computing prosodic cues are connected to, and in some cases partially overlap with the regions controlling aggressive responses (see Miller et al., 2008 for details). As with grammar, one could expect a feedback effect between the control of prosody and the control of aggression. More specifically, since increased HSD essentially resulted in more cortical control on selected subcortical areas, the emergence of a true linguistic prosody might have resulted from increased top-down effects of the language-related areas on subcortical areas as we evolved more prosocial (consider that affective intonation is more dependent on subcortical regions, whereas linguistic prosody is more dependent on cortical regions; see Pihan, 2006; Wildgruber et al., 2006 for details). This effect would have been similar to the effect hypothesized for grammar. And in fact, quite an ample overlap exists between phrase-level prosody and syntax, with. e.g., prosodic features marking key sentence constituents and sentence types (Elfner, 2018). Likewise, prosody serves as a scaffolding for the acquisition of grammar (Brooks, 1997).

**Figure 3:**
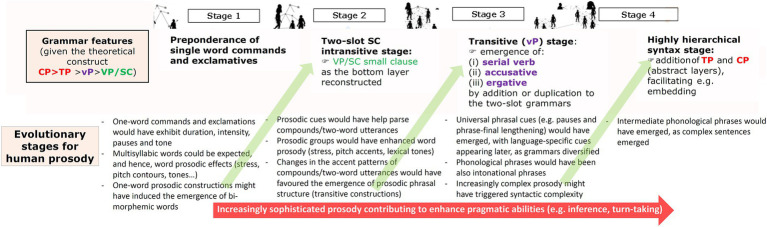
An outline of the evolution of prosody under the effect of HSD (reproduced from Benítez-Burraco and Elvira-García, 2023, Figure 2).

Overall, one could expect that as grammar gained complexity under the effects of HSD, prosody evolved more complex too, but also that the complexification of prosody contributed to make grammar more complex, mostly via the grammaticalization of selected prosodic features, as discussed below. Figure 3 summarizes our proposal. The model encompasses four stages, which parallel the four evolutionary stages hypothesized for grammar.

As noted in subsection 3.1., for Stage 1 we hypothesized simple languages expressing emotions through commands, threats, and exclamations, all of them consisting in single words. Because of physiological reasons, humans cannot produce strings of sounds that lack prosodic features: even the simplest vocalization shows pitch, duration, intensity and pause. In fact, we seem to be biologically programmed to detect differences in pitch contours, prosodic patterns, and stress patterns (Bhatara et al., 2018). Since all world languages convey emotions through changes in pitch and voice quality (Quinto et al., 2013; Wang and Lee, 2014), the languages spoken during this stage can be expected to have exhibited these prosodic features too. For this stage, one could also expect multisyllabic single-word utterances, since syllables and syllable composites have been found in the calls of other mammals, like bats (Chi et al., 2020). The finding that newborns are able to segment utterances into syllables supports this view too (Teinonen et al., 2009; Fló et al., 2019). For such multisyllabic words some sort of stress patterns could be hypothesized as well. Interestingly, in present-day tonal languages, pitch contours (or pitch ranges) can be employed to convey pragmatic meanings, even in the case of single words (Connell et al., 1983). One intriguing possibility is that some of the pitch and voice quality properties of the one-word utterances predicted for this Stage 1 became grammaticalized at some point, first as markers of the type of emotion conveyed by the utterance, but later as true pragmatic markers. Additionally, typological research has found evidence that complex pitch contours can trigger the creation of new morphemes, as observed in the vocatives of several languages (e.g., Sóskuthy and Roettger, 2020). This opens the door to the possibility that during Stage 1 some pitch contours associated to specific pragmatic functions, like warnings, favored the emergence of especific morphemes, thus contributing to the arrival of the two-slot grammars hypothesized for Stage 2 in the model.

As discussed in Section 3.1. for this Stage 2 we have hypothesized the emergence of the first grammars capable of combining single words into compounds. Since word prosody is found in all present-day languages (Nespor and Vogel, 2007) and because the ability to identify words relying on prosodic cues emerges early during ontogeny (e.g., Shukla et al., 2007; Bosch et al., 2013), the existence of a true word prosody can be confidently hypothesized for this Stage 2. At the same time, we expect that word prosody also contributed to the sophistication of grammar, not only to the emergence of the two-slot grammars hypothesized for this stage, as noted, but also to the transition to the three-slot grammars predicted for Stage 3. Notice that in present-day languages, asymmetric compounds consisting of a head and a dependent typically experience a loss of stress (see Hualde, 2007 and Rao, 2015 for Spanish; or Liberman and Sproat, 1992 for English). Accordingly, during Stage 2, some compounds might have experienced a similar loss of stress, this contributing to the advent of true prosodic phrasal markers and structures, which could have helped the development of intransitive constructions, the hallmark of the languages spoken during Stage 2. Nonetheless, by the same reasons, this generalization of prosodic phrasal patterns could have facilitated the emergence of the transitive constructions predicted for the next stage, as we discuss below.

For Stage 3, we predicted the advent of the first hierarchical grammars expressing transitivity. The emergence of these grammars should have been paralleled by the emergence of the type of prosodic cues that present-day languages use for marking transitive sentences, like pauses between main constituents, or a pitch downstepping at the end of the utterance (Gussenhoven, 2002). As noted above, a scaffolding for these more complex prosodic markers might have been the type of phonological phrases emerged during Stage 2 from the prosodic reanalysis of compounds. In turn, a richer prosodic marking might have bootstrapped the more complex syntactic constructions hypothesized for this Stage 3, as observed during language acquisition by the child (Brusini et al., 2018; de Carvalho et al., 2018). That said, prosodic cues might have become more diverse and complex during this Stage 3 because pragmatics complexified during this stage too. As noted in Section 3.1, these Stage 3-languages can be roughly regarded type-S languages. But remember from Section 2, that type-S languages exhibit a notable contextual dependency, with a great amount of meaning being conveyed through idioms, implicatures, and references to the shared knowledge or the common ground. Prosody plays an important role in all this, particularly in distinguishing given or known information from new or pragmatically relevant information, usually through specific pitch patterns (Dennison and Schafer, 2010; Huang and Snedeker, 2018; Roettger et al., 2019). As we will discuss in detail in Section 3.4 below, HSD, which reached its peak during this period, might have contributed in more direct ways to the potentiation of our inferential abilities and to our advanced pragmatic capacities, more generally, through selected impacts on our cognition and behavior. To finish, as also suggested for Stages 1 and 2, this more complex prosody can be expected to have contributed to the sophistication of grammar, specifically to the generalization of embedding, and ultimately to the advent of the sort of languages hypothesized for Stage 4. One reason is that, as observed in some languages like Mohawk, at some initial point prosody can be the only marker of embedding, with full-fledged complement constructions appearing later (see Mithun, 2009 for discussion). Another reason is that prosody usually helps disambiguate between dependent and independent clauses when segmental contents are ambiguous (see Elvira-García et al., 2017 for discussion). Arguably, recursion could have been used during earlier stages, as in rhythmic patterns (morae, syllables or feet), or even in compounding, but our view is that it was only fully exploited at the sentence level during Stage 4.

Notice that in the discussion above we have mostly focused on upper-level categories (like phonological words, phonological phrases, intonational phrases and whole utterances), while we have largely ignored lower-level components, particularly rhythmic categories (like feet, syllables, or morae). The reason is that the latter can be expected to be evolutionarily older, since they have been found in many other species (see, e.g., Mann et al., 2021 on consonant-like and vowel-like sounds in birds).

### 3.3 HSD and semantics

There is ample evidence that other species can acquire and use symbols for referring to specific aspects of the environment, particularly, if properly trained (Seyfarth et al., 1980; Krause and Beran, 2020). Species closer to us can also use gestures in a context-dependent way for conveying different meanings according to the setting and/or the interlocutor, similarly to how humans use words (Hobaiter et al., 2022). Thinking of extinct hominins, one could thus safely hypothesize that they owned ampler and richer “vocabularies” compared to present-day primates, not only because of their bigger brains, but also because they exhibited more complex behaviors, social lives, and interactions with their environment. It is far beyond the scope of this review paper to discuss the purported features of other hominins’ lexicons. Our interest is put instead on the narrower question of the potential impact, if any, of HSD on the semantics of the different languages spoken by anatomically-modern humans (henceforth, AMHs), with a focus on how their lexicons might have changed over time. Being necessarily speculative, the view that HSD did contribute to the diversification of the vocabularies of the languages spoken by our ancestors is not totally baseless. Because of the behavioral and cognitive changes brought about by HSD, of the sort discussed in previous subsections, one could argue that HSD favored, specifically, three processes that make vocabularies more complex: conceptual blending, categorization, and grammaticalization.

As noted enough, HSD seemingly enhanced our cross-modal thinking, which enables us to combine concepts belonging to different core knowledge systems. This can be expected to have enlarged the vocabularies of the first languages spoken by AMHs, particularly, the number of words denoting concepts without a real correlate (e.g., ‘lion-man’). Moreover, some preexisting words might have enriched their meanings by gaining connotative senses, particularly, synesthetic values (i.e., idiosyncratic associations with other perceptual domains). The main reason is that synesthesia is a type of cross-modality, or more properly, as Cuskley and Kirby (2013: 871) puts it, of super cross-modal association.

Furthermore, as we reasoned in a recent paper (Benítez-Burraco et al., 2023), increased cross-modality might have contributed as well to the improvement of our categorization abilities, which are also relevant for vocabulary building. In truth, we have advocated for a feedback loop between the sophistication of our categorization abilities and the gradual emergence of syntactic structure, including Merge, of the sort discussed in Section 3.1 above. Our hypothesis is that our enhanced categorization abilities resulting from our increased cross-modal capability did not only result in more diverse categories, but also in more tokens within each category. Both types of diversity are necessary for Merge to take off in a systematic and productive way. In turn, this potentiation of our combinatorial abilities can be expected to have improved our categorization abilities, because of such an increase in the number of both categories and items within each category.

Improved categorization abilities certainly enable us to understand (and describe) the world in more accurate ways. Having said that, one type of categorization concerns word classes, which are a core structural aspect of languages. Over time, new grammatical categories can emerge though grammaticalization. Heine and Kuteva (2007) has proposed an appealing evolutionary model for language according to which the diversity of word classes, as found in present-day languages, might have resulted from labels for things, in essence, from noun(−like) symbols. Our contention here is that our increased HSD might have favored this trend too. Two lines of reasoning (discussed in depth in Benítez-Burraco, 2017) support this view. First, grammaticalization heavily depends on cognitive abilities like inferencing, metaphorization, or metonymization that were seemingly potentiated by our enhanced cross-modal thinking, in turn resulting from our increased HSD. Second, grammaticalization is also triggered by social factors/needs, like expressing new types of social bounds, or creating new fashionable expressions (see Heine and Kuteva, 2007: 323–329 for details). The richer social environment and the increased sensibility to social cues brought about by our HSD should have favored this trend too.

One example of the emergence (or at least the spread) of one word class under the effects of HSD concerns ideophones. Ideophones are marked words that depict, in vivid and conventionalized ways, sensory images or events (Dingemanse, 2012, 2018, 2019). They are a word class in many languages, and are endowed with distinctive phonological, semantic, morphosyntactic, and pragmatic properties; but overall, they can be regarded as sound-symbolic words, and particularly, synesthetic words. In a recent paper (Di Paola et al., 2024), we have argued that because of this synesthetic-like nature, ideophones could not be very old, while at the same time, might have been more frequent in the past than nowadays. Specifically, and in line with our evolutionary model for language under the effects of HSD, we suggest that ideophones emerged (or at least, generalized) during Stage 2, but became less abundant during Stage 4. The main reason is that synesthesia(−like) effects demand a potentiated cross-modality, which, as discussed enough, might have emerged progressively under the effect of our HSD, which reached its peak during Stage 3. Supporting this view, synesthetes show increased abilities for understanding unfamiliar sound-symbolic words (Bankieris and Simner, 2015). As with other aspects of language, we support the view that the emergence of ideophones might have fostered the complexification of language, mostly because ideophones often come in reduplicative pairs (Dingemanse, 2019), so that they might have scaffolded the two-slot grammars hypothesized for Stage 2.

### 3.4 HSD and pragmatics

To finish this detailed characterization of the evolution of language under the effects of HSD, we will now provide some reasons why HSD might have contributed as well to the advent of modern uses of language, that is, modern pragmatics. To some extent, our pragmatic abilities can be expected to have improved as more elaborated grammatical, prosodic, and lexical resources emerged under the effects of HSD, as discussed in previous subsections. A more sophisticated grammar and a richer vocabulary allow the expression of one’s thoughts in more precise ways, and thus the better defense of one’s beliefs against the beliefs of others, this contributing to the optimization of persuasive reciprocity, as it is typically found in human conversations. Likewise, a richer prosody typically results in more abundant and varied pragmatic markers. In turn, as also argued in subsections 3.1 to 3.3, this improvement of our pragmatic capabilities might have helped the sophistication of the structural aspects of language. Nonetheless, in a recent paper (Benítez-Burraco et al., 2021a), we have argued that the potentiation of pragmatics might have also been a direct outcome of the behavioral and cognitive changes brought about by HSD, resulting in more sophisticated turn-takings, as well as more complex inferential capabilities, which are key aspects of our pragmatic abilities. Among the behavioral changes, two of them stand as particularly relevant. First, prolonged face-to-face interactions. Second, more cooperation and increased sensitivity to the needs of one’s interlocutor. Both are at the heart of fine-tuned turn-taking. With regards to the cognitive changes, the most important one was likely the full emergence of our social brain. This has been claimed to result from the generalization of pair-bonds to other, non-reproductive relationships (Dunbar, 2009), and/or the potentiation of our evolutionary tendency toward social dependency for survival (Atzil et al., 2018). Both trends can be safely expected to have been fostered by a reduction in reactive aggression. However, the potentiation of cross-modal thinking, as we characterized it earlier, was relevant too, since cross-modality is central to figurative uses of language, such as metaphors and metonyms, and particularly, to pragmatic inferencing. The ultimate consequence of all these behavioral and cognitive changes was, we contend, that face-to-face interactions became more frequent and richer, with richer inferences and with more complex meanings being conveyed by more indirect means.

## 4 Conclusions and future prospects

In this review paper, we have supported the view that both the structural and the functional aspects of language might have co-evolved gradually in AMHs under the effects of HSD, with changes in aggression types and levels, and in language structure and use being intertwined in a complex feedback loop. Certainly, HSD is not the only factor accounting for the emergence of our distinctive linguisticality and the type of languages we speak nowadays, but it seems to be an important one. Compared to other evolutionary models for language, one strong point of this HSD account is that it acknowledges a stronger continuity between our linguisticality and the cognitive abilities and behaviors exhibited by other species. A second strong point is that it grants cultural niche construction, cultural evolution, and gene-culture co-evolution a more central role than others in the advent of modern languages.

There are several lines of (ongoing) research that could help test and improve this HSD approach to language evolution.

### 4.1 The timeline of HSD

One first aspect of interest is the timing and the presentation of the HSD phenotype during human history. As noted already several times, there is evidence that some of the physical features of HSD increased over time in our species, reaching its peak during Upper Paleolithic. It is certainly of relevance to know more about the starting point of the HSD process, the environmental factors that triggered and fostered it, and the precise stages it followed. Likewise, it is worth clarifying if other hominin species (particularly, Neanderthals) also went through a HSD process, even if less markedly, since there is evidence that other primates, specifically bonobos, have been self-domesticated (Hare et al., 2012). Neanderthals had quite rich social lives, but also exhibited reduced contacts with non-kin people (Skov et al., 2022; Slimak et al., 2024). Additionally, their physical features are only partially compatible with a self-domesticated phenotype. And they showed violence levels quite similar to those of early AMHs (Zollikofer et al., 2002). One promising way of addressing this question is delving into the molecular mechanism of HSD. In animals, there is evidence that domestication entails (and is promoted by) selected genetic and epigenetic changes. Past research by Theofanopoulou et al. (2017) found a statistically significant overlap between genes showing evidence of selective sweeps in AMHs compared to Neanderthals and Denisovans, and genes selected in several domesticated species, particularly the dog, the cat, the horse, and the taurine cattle. However, this research involved present-day human genomes, so it was essentially inconclusive about the timing of the selection events. In turn, Benítez-Burraco et al. (2021b), using an improved list of genes selected in domesticated varieties of mammals, as well as European genomes from Late Neolithic/Bronze Age, found that candidates for mammal domestication have been accumulating nonsynonymous mutations during the past 6.000 years. This is a period when important changes in human behavior and culture occurred (including the spread of agricultural practices, sedentism and urbanization), when population density increased notably, and when long-distance trading routes developed. These changes reshaped not only the gene pool of Europe, but also modified its linguistic landscape, resulting in the nearly total replacement of the European hunter-gatherer languages by Indo-European languages. One possibility discussed by these authors is that the observed changes in genes related to HSD are a hallmark (and perhaps favored in some way) the emergence of proactive aggression within these populations and the transition from Type S-languages to Type X-languages in Europe. By the reasons discussed in section 2, acquiring a Type X- language might demand some cognitive adaptation, like the improvement of procedural memory abilities because of their more complex syntax. Interestingly, in these European samples from 6.000 years ago, previous research had found evidence of selection in two pathways related to cognition, particularly, to long-term potentiation, which underlies synaptic plasticity and ultimately, memory and learning abilities (Chekalin et al., 2019). Interestingly too, the domestication candidates showing signals of selection in AMHs compared to Neanderthals and Denisovans, as listed by Theofanopoulou and colleagues, do not overlap with the candidates showing signals of selection in Europeans during the last 6,000 years, as identified by Benítez-Burraco and colleagues. One possible explanation for this is that the former genes account for the milder HSD phenotype exhibited by early AMHs, particularly during Stages 1 and 2 in our model, whereas the latter genes are responsible for more recent HSD traits, as observed during Stage 3 and the transition to Stage 4.

### 4.2 Animal self-domestication and communication

A second line of research concerns the potential impact of self-domestication on the communication abilities of other species. As noted above, several species others than humans have been claimed to have gone through a self-domestication process. Bonobos are an outstanding example. Compared to chimpanzees, the structure of their vocalizations seems more complex (Taglialatela et al., 2018). Also, they use more indexical cues and acquire better linguistic skills in experimental settings (Savage-Rumbaugh et al., 1996; Gillespie-Lynch et al., 2014; MacLean and Hare, 2015). And there is evidence of a multimodal use of socially-directed calls by bonobos, but not by chimps (Genty et al., 2014). It would be interesting to prove that these distinctive features in the domain of communication correlate with (and can be explained by) the physical, behavioral, and cognitive changes brought about by their self-domestication, similarly to what we have hypothesized in Section 3 for human language. Genetic studies comparing the sequences of candidate genes for mammal domestication in bonobos and chimps would be also worth conducting. Likewise, it would be interesting to check whether the opposite is also true, namely, that species showing abilities that are crucial for developing complex communicative signals also exhibit signals of self-domestication. Actually, this seems to be the case. In an ongoing research, De Reus et al. (2024) have found that vocal learning mammals show most of the behavioral and cognitive hallmarks of self-domestication, including increased prosociality, exploratory behavior, and play behavior; more social tolerance; and sophisticated communication and information sharing abilities. Vocal learning is, of course, a prerequisite for human speech, and a key ability underlying language acquisition and development by children. Additional support to this view results from the examination of elephants. Elephants are well known for their advanced social skills, but they are also vocal leaners. In a recent paper, Raviv et al. (2023) found that, among other features, elephants exhibit reduced reactive aggression (the triggering factor of domestication and self-domestication), cortisol homeostasis sensitive to social factors (with changes in cortisol levels being a reliable biomarker of reactivity to stress), and an extended juvenile period (associated with increased exploratory play and resulting in enhanced social learning). Interestingly, when they compared the set of genes showing evidence of positive selection in elephants with the set of genes involved in mammal domestication, they found nearly 40 overlapping genes. According to the authors, several of them stand out as potential causative factors for some of the self-domestication features observed in elephants, as in humans they are related to communication problems, social and behavioral disturbances, or an altered presentation of HSD features. Interestingly too, they also found that the genes that have been positively selected in the elephant lineage are enriched in pathways likely related to domestication. Specifically, they observed enrichment in pathways involved in socialization and the management of aggression, including serotonin signaling and corticotropin signaling, the latter playing a key role in stress responses.

### 4.3 Feralization and communication

A third promising line of research concerns feral animals. Feralization is the process by which a once-domesticated animal returns to a wild-like state due to desocialization from humans, or the absence of human socializing pressures. Whereas domestication mostly results from selection for tameness, feralization usually involves the reactivation of mechanisms triggering reactive aggression. As discussed in detail by Niego and Benítez-Burraco (2022), not all domestication features are lost in feral animals, but some of them (particularly, prosocial behavior) are indeed reversed, seemingly because these traits are more sensitive to environmental changes. Significantly, the communication patterns and abilities exhibited by feral animals are halfway between those showed by domesticated animals and the ones observed in their wild counterparts. Typically, their communicative signals are less diverse and versatile, and are used in less varied social situations compared to domestic animals. In their paper, Niego and Benítez-Burraco also compared the set of genes under positive selection in domesticated mammals with the set of genes positively selected in feral animals. Similarly to what can be observed at the phenotypic level, only a subset of candidate genes for domestication seems to be involved in feralization. Potentially, these genes could be related to the features of domestication (and of HSD) that are more sensitive to environmental factors.

### 4.4 HSD and language impairment

A fourth aspect of particular interest is the manifestation of features of HSD in cognitive disorders entailing language deficits. A strong link seems to exist between human evolution and human-specific diseases/conditions, with recently evolved phenotypic traits being more sensitive to developmental perturbations because of the reduced resilience exhibited by their biological components (see Gibson, 2009; Gibson and Lacek, 2020; Pattabiraman et al., 2020 for general discussions). Since HSD seems to be a recent human trait, one would expect that it is also particularly sensitive to developmental damage. Actually, this seems to be the case. Consider the case of autism spectrum disorders (ASD). These are notably prevalent conditions in all human populations, present-day and past populations, and entail diverse behavioral and cognitive disturbances (Bailey et al., 1996; Frith and Happé, 2005; Lord et al., 2020). These include problems with structural aspects of language, but also with figurative language, language use in conversational settings, and pragmatics more generally (Rapin and Dunn, 2003; Tager-Flusberg et al., 2005; Tager-Flusberg, 2006; Eigsti et al., 2007). Research has found ample evidence that candidate genes for ASD are enriched in genes positively selected in AMHs after our split from extinct hominins (Polimanti and Gelernter, 2017), supporting the link between human cognitive and behavioral evolution and human cognitive and behavioral impairment. Research by Benítez-Burraco et al. (2016) found, specifically, that features of HSD present attenuated in people within the ASD spectrum. Likewise, many candidates for mammal domestication are among the candidates for ASD and/or exhibit altered expression profiles in the brain of people with these conditions, whereas many candidate genes for ASD show signals of positive selection in domesticated animals.

Different hypotheses about the etiology of ASD have been formulated to date, including an altered Theory of Mind, a reduced sensitivity to social relations and social cues, or the effect of selected environmental factors on brain development (see discussions by Bölte et al., 2019; Steinman, 2020; Yoon et al., 2020; or Sauer et al., 2021, among many others). The same happens, specifically, with the language problems observed in autists, which have been claimed to result from diverse cognitive, behavioral, or even motor underlying deficits (Walenski et al., 2006; Preissler, 2008; Lindgren et al., 2009; Belmonte et al., 2013; Lampri et al., 2024). In a recent paper (Benítez-Burraco and Progovac, 2023), we have hypothesized that the language deficits typically observed in ASD, both structural and functional, might result, at least in part, from the alteration of the biological mechanism underlying HSD. Besides the deficits mentioned above, people within the ASD spectrum also feature an increased aptitude for rule-governed abilities, including language (Baron-Cohen et al., 2009; Ward et al., 2017). This seemingly explains their typically hyper-systemizing behavior, as observed, e.g., in their frequent over-regularization of past-tense forms (Baron-Cohen et al., 2009). Overall, the ASD phenotype could be characterized as “rigid”: a rigid behavior, a rigid application of grammar rules, a rigid interpretation of non-literal language. As we discuss in the paper, this “rigidity” is suggestive of an increased striatal function. Nonetheless, typical language processing (and acquisition) results from a delicate balance between the application of rules and patterns (i.e., rigidity), and the ability to suspend such rules when exceptions need to be learnt or when metaphorical extension is needed to understand utterances (i.e., flexibility), with this balance resulting from the intervention of cortical areas on subcortical function (see Benítez-Burraco and Progovac, 2023 for details). Remember from subsection 3.1, that a functional connection, and seemingly a partial overlap exists between the brain mechanisms involved in the modulation of aggression and the mechanisms supporting language. In that subsection, we also highlighted that the feedback loop between HSD and language supported by this partially common neural substrate and resulting in richer language structures and richer pragmatic abilities might have involved an increased cortical control over selected subcortical areas. Accordingly, our proposal about the etiology of language dysfunction in ASD is that the “linguistic rigidity,” and ultimately the increased striatal function and the reduced connection with the cortex found in this condition, could be a consequence of the abnormal presentation of HSD features. In our view, this possibility is supported by the finding that in other conditions resulting from striatal damage (like Tourette’s syndrome) patients also exhibit rigidity with syntactic rules, as well as problems with figurative language (Eddy et al., 2010; Drury et al., 2018), but also increased reactive aggression (Ganos et al., 2014).

If our hypothesis is correct, it should not only help improve our understanding of the prevalence, etiology, and symptomatology of ASD, but also clarify the evolutionary history of the human linguisticality, because of the link between human disease and human evolution. Very broadly speaking, ASD would result from the attenuation of otherwise beneficial traits for language acquisition and use that were potentiated by our HSD, including enhanced cross-modal thinking and enhanced rule-governed systematicity (important for the evolution of the cognitive hardware of language), as well as enhanced control of aggression (important also for socialization and the cultural evolution of language). ASD could be further construed as a window to the earliest stages in the complexification of languages in our species under the forces of HSD, which featured a high degree of reactive aggression (and thus, a low degree of sociability), together with a diminished cross-modality (and thus, reduced metaphoricity and merging abilities). We have equally reasoned that another prevalent, human-specific cognitive disorder like schizophrenia (SZ) might provide a glimpse into a somewhat later stage in the complexification of human languages, when the higher disinhibited connectivity in the brain networks highlighted before would have resulted in exaggerated, super cross-modality (Stage 3 in our model) (see Benítez-Burraco and Progovac, 2021 for details). The reason is that in people with SZ, features of HSD are presented exaggerated at the anatomical, physiological, and behavioral levels (Benítez-Burraco, 2017). Likewise, nearly 20% of the candidate genes for mammal domestication are candidates for this condition, with around 75% of them being also differentially expressed in brain areas like the frontal cortex, the associative striatum, and the hippocampus, which exhibit structural and functional anomalies in schizophrenics, play a role in language processing, and show differences in domesticated animals compared to their wild conspecifics (see Benítez-Burraco, 2017 for detailed discussion).

### 4.5 Animal models of HSD

An additional line of research aimed to provide support to the HSD hypothesis of language evolution concerns the development of animal models of HSD. Animal models allow to study in controlled ways the effects of the myriads of factors involved in development and disease (Phillips and Roth, 2019). Mammals that are known (or hypothesized) to have gone through a self-domestication process (like bonobos or elephants) are not idoneous for this type of research. In a recent paper (Anastasiadi et al., 2022), we have proposed to use the sea bass as an animal model for the genetic, and particularly the epigenetic changes underlying HSD. Sea basses can be bred in captivity very easily and we already have a good understanding of the genetic and epigenetic changes occurred during their domestication, with these changes parallelling the changes observed in domesticated mammals (Anastasiadi and Piferrer, 2019). Certainly, epigenetics can be expected to have played a crucial role in HSD, since epigenetic mechanisms are key to integrate environmental information and ultimately, to generate plastic responses to environmental changes. Most differences between AMHs and extinct hominins indeed pertain to the epigenome, with most of them affecting body parts known to be impacted by HSD and involved in language, particularly, the face (Gokhman et al., 2020). Moreover, epigenetic dysregulation is widely acknowledged as a key etiological factor of cognitive disorders (Gräff and Mansuy, 2009; Peedicayil and Grayson, 2018). However, an important limitation of current attempts to determine the impact of epigenetic changes in HSD is that we have access to fossil bones only. But epigenetic signals are different in the different body tissues. And in the case of HSD, soft tissues like the brain or the organs derived from the neural crest are more relevant than the bones. This is another reason to rely on animal models. In our research, we performed a comparison between known candidates for mammal domestication and genes differentially methylated in the domesticated sea bass. The significant overlap we found reinforces the view that the sea bass is a good animal model for mammal domestication, and arguably, for HSD too. Additional support to this view comes from our other finding, namely that a significant overlap exists between the genes showing epigenetic changes in early domesticates of the European sea bass and the genes exhibiting methylation changes in AMHs compared to Neanderthals and Denisovans. The overlapping genes are involved in processes like limb morphogenesis and in phenotypes like abnormal jaw morphology and hypopigmentation, which are related to the changes observed during domestication (and HSD). Finally, we found a significant overlap between the genes exhibiting epigenetic changes in early domesticates of the European sea bass and genes showing methylation changes in the brain of people with SZ as well as in the brain and the blood of subjects with ASD. Overlapping genes are involved in processes such as neural crest differentiation and ectoderm differentiation. This is suggestive of the sea bass also being a good model of human cognitive conditions entailing, and seemingly resulting from an abnormal HSD process.

### 4.6 HSD and language diversity

A final line of inquiry of potential interest for testing this HSD account of language evolution pertains the domain of linguistic typology. By the reasons provided in section 2, the languages spoken by present-day isolated human groups can be roughly characterized as strongly Type S-languages. As also noted in that section, such languages can be regarded as rough proxies for the languages spoken during Upper Paleolithic, particularly during Stage 3 in our model, when the effects of HSD seemingly reached their peak. Interestingly, some HSD features have been found to exhibit a variable presentation in present-day human populations (Gleeson and Kushnick, 2018). This is not surprising, since HSD is above all a response to changes in the human social environment. By all these reasons, it would be interesting to check whether, globally, speakers of Type S-languages (and more generally, users of esoteric communication) show more marked features of HSD, for example, a stronger feminization of the skull, as found in Upper Paleolithic populations (Cieri et al., 2014).

## Data Availability

The original contributions presented in the study are included in the article, further inquiries can be directed to the corresponding author.
