# Corrigendum: How (and why) languages became more complex as we evolved more prosocial: the human self-domestication view

**DOI:** 10.3389/fpsyg.2025.1583411

**Published:** 2025-03-11

**Authors:** Antonio Benítez-Burraco

**Affiliations:** Department of Spanish, Linguistics and Theory of Literature (Linguistics), Faculty of Philology, University of Seville, Seville, Spain

**Keywords:** language evolution, language structural complexity, language uses, prehistory, aggression, human self-domestication

In the published article, there was an error in the Funding statement and some funding information was omitted. Original Funding statement:

The author(s) declare that financial support was received for the research, authorship, and/or publication of this article. This research was supported by grant PID2023-147095NB-I00 funded by MCIN/AEI/10.13039/501100011033.

The correct Funding statement appears below.

The author(s) declare that financial support was received for the research and/or publication of this article. This research was supported by grant PID2023-147095NB-I00 funded by MCIN/AEI/10.13039/501100011033 and by ERDF/EU.

The author apologizes for this error and state that this does not change the scientific conclusions of the article in any way. The original article has been updated.

